# Multiplex qPCR for Detection and Absolute Quantification of Malaria

**DOI:** 10.1371/journal.pone.0071539

**Published:** 2013-08-29

**Authors:** Edwin Kamau, Saba Alemayehu, Karla C. Feghali, David Saunders, Christian F. Ockenhouse

**Affiliations:** 1 Military Malaria Research Program, Malaria Vaccine Branch, Walter Reed Army Institute of Research, Silver Spring, Maryland, United States of America; 2 Department of Immunology and Medicine, US Army Medical Corps, Armed Forces Research Institute of Medical Sciences (USAMC-AFRIMS), Bangkok, Thailand; Institut national de la santé et de la recherche médicale - Institut Cochin, France

## Abstract

We describe development of an absolute multiplex quantitative real-time PCR for detection of *Plasmodium* spp., *P. falciparum* and *P. vivax* targets in order to produce an assay amenable to high throughput but with reduced costs. Important qPCR experimental details and information that is critical to performance and reliability of assay results were investigated. Inhibition studies were performed to test and compare co-purification of PCR inhibitors in samples extracted from whole blood using either the manual or automated methods. To establish the most optimal qPCR reaction volume, volume titration of the reaction master mix was performed starting at 10 µl to 1 µl reaction master mix with 1 µl of template DNA in each reaction. As the reaction volume decreased, qPCR assays became more efficient with 1 µl reaction master mix being the most efficient. For more accurate quantification of parasites in a sample, we developed plasmid DNAs for all the three assay targets for absolute quantification. All of absolute qPCR assays performed with efficiency of more than 94%, R^2^ values greater than 0.99 and the STDEV of each replicate was <0.167. Linear regression plots generated from absolute qPCR assays were used to estimate the corresponding parasite density from relative qPCR in terms of parasite/µl. One copy of plasmid DNA was established to be equivalent to 0.1 parasite/µl for *Plasmodium* spp. assay, 0.281 parasites for *P. falciparum* assay and 0.127 parasite/µl for *P. vivax* assay. This study demonstrates for the first time use of plasmid DNA in absolute quantification of malaria parasite. The use of plasmid DNA standard in quantification of malaria parasite will be critical as efforts are underway to harmonize molecular assays used in diagnosis of malaria.

## Introduction

Malaria remains one of the most burdensome and lethal infectious diseases in tropical and sub-tropical countries. Despite gains made in diagnosis of malaria by use of molecular methods, microscopy remains the gold standard technique for diagnosing and quantifying malaria. However, microscopy has many limitations such as the need for the extensive training, inter-observer variability, difficulty in certifying results, and low sensitivity [1], [2]. Quantitative Real-time PCR (qPCR) is now commonly used as a confirmatory method for malaria diagnosis especially in clinical trials and in reference laboratories where precise quantification is critical [3], [4], [5]. Although use of PCR for diagnosis of malaria was first published in 1990 [6], it was not until 2001 and 2002 that the application of qPCR in diagnosis of malaria was first described [7], [8]. PCR offers several advantages over microscopy in diagnosis of malaria in several regards. First, PCR is both highly sensitive and highly specific allowing explicit identification of malarial species [9], [10]. PCR can also be used for precise parasite quantification through qPCR methods. Relative quantification is used in qPCR where the parasite density is first determined by microscopy. Serially diluted DNA from the sample with known parasite density is then used as a standard to determine parasite density of the unknown [3], [4], [5], [6], [7], [8], [11].

Absolute quantification uses a calibration curve where known amounts of external targets are amplified in a parallel group of reactions run under identical conditions to that of the unknown samples. The standard molecules such as recombinant plasmid DNA carrying the target gene (plasmid DNA), genomic DNA or commercially synthesized oligonucleotide can be used. Among the various types of standard DNA available, plasmid DNA is most commonly chosen due to its high stability and reproducibility [12]. The absolute quantities of the standard DNA must first be determined by some other independent means such as UV absorbance (OD_260_) or fluorescent dye-binding methods. The concentration of the DNA is then converted to the number of copies or Genomic Equivalence [GE] using DNA molecular weight. Absolute qPCR is used to determine the quantity of the unknown based on linear regression calculations of the standards. Absolute quantification has several advantages over relative quantification; it is highly reproducible, allows the generation of highly specific, sensitive and reproducible data [13]. It is a more precise approach for analyzing quantitative data and requires minimal amount of optimization and validation. Absolute quantification of *Plasmodium* by qPCR has not been described.

In this study, we describe an absolute quantitative multiplex qPCR assay for detection of *Plasmodium* spp., *P*. *falciparum* and *P. vivax* parasites. The absolute quantification is reported as parasites/µl, the same units as those used in microscopy.

## Materials and Methods

### Ethics

Clinical samples used in this study were obtained either from Kenya [*P. falciparum*] or Cambodia [*P. vivax*]. The Kenyan samples were from a Phase IIb pediatric clinical trial conducted between March 2005 and April 2006 at the KEMRI/Walter Reed Project, Kombewa Clinic in the Kombewa Division of Kisumu District, Nyanza Province, Western Kenya. The trial registration for this study can be found at clinicaltrials.gov, identifier NCT00317473. The details of this study have also been published elsewhere [14]. The study was approved by Ethical Review Committee of the Kenya Medical Research Institute, Nairobi, Kenya. The Cambodian samples were from a study conducted in 2010 in Battambang and Oddar Meancheay Provinces along the Thai border. The details of this study have been published elsewhere [15]. This study was approved by the National Ethical Committee for Health Research, Phnom Penh, Cambodia. Both studies were also approved by the Walter Reed Army Institute of Research (WRAIR) Institutional Review Board, Silver Spring, Maryland, USA and by the Human Subjects Research and Review Board of the Surgeon General of the U.S. Army at Fort Detrick, Maryland, USA. The Cambodia study was conducted under approved protocol WRAIR 1576. Protocols used in these studies complied with International Conference on Harmonization Good Clinical Practice (ICH-GCP) guidelines. These studies were conducted in accordance with the principles described in the Nuremberg Code and the Belmont Report including all federal regulations regarding the protection of human participants as described in 32 CFR 219 (The Common Rule) and instructions from the Department of Defense and the Department of the Army. They also followed the internal policies for human subject protections and the standards for the responsible conduct of research of the US Army Medical Research and Materiel Command. WRAIR holds a Federal Wide Assurance from the Office of Human Research Protections under the Department of Health and Human Services. All key study personnel in both studies were certified as having completed mandatory human research ethics education curricula and training under the direction of the WRAIR IRB Human Subjects Protection Program. All potential study subjects provided written informed consent before screening and enrollment and had to pass an assessment of understanding.

### Clinical Samples

For assessment of malaria, a peripheral blood smear was obtained from subjects who presented to the study sites with fever or a history of fever within 48 h or an illness that the attending doctor suspected might be due to malaria infection. After Giemsa staining, thin and thick blood smear slides from each sample were independently examined by two or three expert microscopists for detection of *Plasmodium* and counts where applicable. All malaria microscopists were fully trained and were required to pass a competency and proficiency test prior to reading slides for the study. The parasite density presented in this study is the average density obtained by the independent (blinded from each other’s results) microscopists. Blood samples obtained from these studies were stored frozen in −20°C until needed. Genomic DNA was extracted from the whole blood either manually using the QIAamp DNA Blood Mini Kit or automated with the EZ1 DNA blood kit on the EZ1 Advanced XL automated sample purification system (Qiagen, Valencia, CA) as recommended by the manufacturer. The DNA from the two studies was extracted at different time points; the DNA from the Cambodian trial was extracted when this study was being conducted, but the DNA from the Kenyan trial was extracted 5–6 years ago. The extracted DNA was stored in −20°C until needed.

### *Plasmodium Falciparum* Reference Reagent

The WHO international standard for *P. falciparum* DNA nucleic acid amplification technology (NAT) assays, obtained from the National Institute for Biological Standards and Control (NIBSC; Hertfordshire, United Kingdom) was used as the calibration reference reagent for of the *Plasmodium spp*. and *P. falciparum* assays. The standard consists of a freeze-dried preparation of whole blood collected by exchange transfusion from a patient infected with *P. falciparum*. Following NIBSC recommendations, this lyophilized material was suspended in 500 µl of sterile, nuclease-free water to a final concentration of 1×10^9^ IU/ml, which corresponds to a parasitemia of 9.79 parasites/100 red blood cells [11]. The parasite density of the NAT assays after reconstitution was estimated to be 469,920 parasites/µl, based on the average red blood cell count [from uninfected donor] of 4.8×10^6^ RBC/µl. Unless otherwise indicated, fresh uninfected whole blood was used as a diluent to prepare serial dilutions. The uninfected whole blood was obtained from donors from Washington DC metropolitan area under WRAIR approved protocol. After reconstitution, genomic DNA was extracted with the EZ1 DNA blood kit on the EZ1 Advanced XL automated sample purification system (Qiagen, Valencia, CA) as recommended by the manufacturer.

### Primers and Probes Design

Primers and probes for detection of *Plasmodium* spp. and *P. falciparum* have previously been described [5], [16]. Primers and probes for detection of *P. vivax* and RNaseP genes were designed using Primer Express 3.0 software (Applied Biosystems, Foster City, CA) after the alignment of available GenBank sequences for the *P. vivax* 18S rRNA gene, accession number AY579418 and human RNaseP gene, accession number NM_001104546.1. Fluorophores chosen for each assay were carefully selected and each combination extensively tested to allow optimal performance of the multiplex assay. Table 1 show primer and probe sequences, fluorophores and the length of primers and probes used in this study. Probes for *P. falciparum*, *P. vivax* and RNaseP assays contained minor groove binder (MGB) groups which form stable duplexes with single-stranded DNA targets, allowing shorter probes to be used for hybridization based assays.

**Table 1:** Primers and probes sequences used for qPCR assays in this study.

Primers Probes	Sequences 5′- 3′	Modifications	Size (bp)
PLU F	GCTCTTTCTTGATTTCTTGGATG		
PLU R	AGCAGGTTAAGATCTCGTTCG		
PLU P	ATGGCCGTTTTTAGTTCGTG	CY5-IB	100
FAL F	ATTGCTTTTGAGAGGTTTTGTTACTTT		
FAL R	GCTGTAGTATTCAAACACAATGAACTCAA		
FAL P	CATAACAGACGGGTAGTCAT	FAM-MGB	95
VIV F	GCAACGCTTCTAGCTTAATCCAC		
VIV R	CAAGCCGAAGCAAAGAAAGTCC		
VIV P	ACTTTGTGCGCATTTTGCTA	VIC-MGB	133
RNaseP F	TGTTTGCAGATTTGGACCTGC		
RNaseP R	AATAGCCAAGGTGAGCGGCT		
RNaseP P	TGCGCGGACTTGTGGA	NED-MGB	84
IC F	AAAGAAACTAGGAGAGATGTGGAACAA		
IC R	AGCTTGGCAGCTTTCTTCTCA		
IC P	ACTGCAGCAGATGACAAGCAGCCCT	CY3-IB	75

Primer and probes for amplification of *Plasmodium* spp., *P. falciparum*, *P. vivax*, RNaseP and internal control (IC) plasmid DNA assays. Sequences for Forward (F), Reverse(R) primers and the Probe (P) are shown.

### Real-time PCR Assays

Amplification and qPCR measurements were performed using the Applied Biosystems 7500 Fast Real-Time PCR System, v 2.0.5 software. The thermal profile used for qPCR if as follows: 5 min at 95°C; 40 cycles of 3 s at 95°C; 30 s 60°C. Each reaction contained 1 µL of template DNA and a reaction master mix containing 1X QuantiFast Probe PCR Master Mix with ROX dye (QIAGEN, USA), 0.4 µM of each primer and 0.2 µM of each probe. All qPCR assays were run with appropriate controls including the Non-Template Control [NTC]. If the assay did not contain DNA or the DNA was below the detection limit, the assay result is denoted as ‘und’ [undetermined].

### Generation of Plasmid DNAs

Primers for *Plasmodium* spp. (PLU), *P. falciparum* (FAL) and *P. vivax* (VIV) assays were used for amplification of PCR fragments from genomic DNA from either *P. falciparum* 3D7 laboratory strain samples or *P. vivax* clinical samples and cloned into TOPO TA vectors. These plasmids are referred to as PLU, FAL or VIV plasmid. To create an inhibition control [IC] plasmid, part of mouse high mobility group protein (HMGB) was cloned into TOPO TA vector. The details of the cloning process and conditions have been previously described [17]. After plasmid DNA carrying the correct clone was purified and tested, the concentration and purity of plasmid DNA was measured using NanoDrop 2000 (Thermo Fisher Scientific Inc, USA) following the manufacturer’s instructions. All DNA samples were required to have a 260/280 ratio of between 1.8 and 2.0. The GE for each assay was calculated using the following equation:
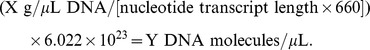



For absolute quantification by qPCR, each plasmid DNA was serially diluted and used in subsequent experiments.

### Relative Standard Curves

Genomic DNA from *P. falciparum* [NAT assays] and *P. vivax* clinical samples were used to generate the relative standard curves for qPCR. For the *P. vivax* clinical samples, expert microscopists determined the parasite density. Five different clinical samples were used to generate relative standard curves for qPCR. Genomic DNA from these samples was extracted using the QIAamp Blood DNA kit (Qiagen, Valencia, CA), serially diluted and used in the relative quantification experiments.

## Results

### Design and Analysis of Multiplex qPCR

A multiplex qPCR assay was designed to simultaneously detect *Plasmodium* spp., *P. falciparum*, *P. vivax* and human RNaseP gene as an endogenous control. These assays are referred to as follows in the manuscript: the *Plasmodium spp.* assay is referred to as PLU assay, the *P. falciparum* assay as FAL assay, and the *P. vivax* as VIV assay. The performance of PLU and FAL assays has been previously described [5]. The sensitivity and specificity of VIV assay was tested using field clinical samples with known parasite densities. To test the analytical sensitivity of the VIV assay, *P. vivax* clinical samples were analyzed using previously published nested PCR assay [18] and then sequenced using standard methods. All nested PCR results and sequences were that of *P. vivax*. To test the specificity of the VIV assay, qPCR experiments were performed using the following non-target agents: *P. ovale*, *P. malariae*, *P. cynomolgi*, *P. knowlesi*, *Babesia microti*, *Trypanosoma cruzi* and *Leishmania*. The VIV assay did not cross-react with any of the non-target organisms tested indicating that the VIV assay have 100% specificity. To test and analyze the assays as a multiplex, genomic DNA containing both *P. falciparum* and *P. vivax*, serially diluted 5-fold to 4 different concentrations was used. The performance of each primer and probe set as a singleplex assay (reaction master mix containing a single set of the primer and probe) and multiplex assay (reaction master mix containing primer and probe sets for all the four targets) was assessed. For the multiplex reactions, analysis was performed for individual targets as well as simultaneous analysis of all the targets. One of the most important features of ABI 7500 system is the ability to scan all the wavelengths during the run and stores this information. After the run is complete, different fluorophores can be selected and re-analyzed. This feature permitted us to run multiplex assays but go back and analyze these assays as multiplex or singleplex. Table 2 shows the average C_T_ values of 5-fold serially diluted genomic DNA, each assay performed in 4 replicates. Data shows that all singleplex and multiplex assays performed the same except for RNaseP assay which performed better in analysis 2. There is no good explanation for this phenomenon since the performance of RNaseP assay is the same for analysis 1 and analysis 3.

**Table 2:** Analysis of the multiplex real-time PCR assay.

Sample Name	Assay Performed	Analysis 1 Mean CT	Analysis 2 Mean CT	Analysis 3 Mean CT
DNA dilution # 1	PLU	19.8	19.85	19.92
DNA dilution # 2	PLU	22.08	22.13	22.01
DNA dilution # 3	PLU	24.38	24.43	24.33
DNA dilution # 4	PLU	26.73	26.78	26.79
DNA dilution # 1	FAL	21.93	21.77	22.08
DNA dilution # 2	FAL	24.28	24.19	24.6
DNA dilution # 3	FAL	26.32	26.3	27.01
DNA dilution # 4	FAL	29.62	29.49	30.06
DNA dilution # 1	VIV	22.17	22	22.22
DNA dilution # 2	VIV	24.9	24.63	24.96
DNA dilution # 3	VIV	27.39	27.08	27.46
DNA dilution # 4	VIV	30.27	29.94	30.05
DNA dilution # 1	RNaseP	25.81	22.5	25.24
DNA dilution # 2	RNaseP	27.79	24.98	27.62
DNA dilution # 3	RNaseP	29.98	27.38	30.29
DNA dilution # 4	RNaseP	33.92	30.31	32.75

Multiplex qPCR assays were performed containing both primer and probe sets for all four targets or primer and probe set for single target. Analysis 1 shows data from multiplex assay analyzed as multiplex where all four targets were analyzed simultaneously. Analysis 2 shows data from multiplex assay but data was analyzed as a single assay for each target. Analysis 3 shows data from single assays.

### Comparison of DNA Extraction Methods and Sample Volume

Genomic DNA was extracted from whole blood either manually using the QIAamp DNA Blood Mini Kit (ME) or automated using the Qiagen EZ1 DNA blood kit (Qiagen, Valencia, CA) on the EZ1 Advanced XL automated sample purification system (EZ). Extraction procedures were performed as recommended by the manufacturer. Samples used in these experiments were prepared by adding *P. falciparum* and *P. vivax* clinical sample into uninfected fresh whole blood. Genomic DNA was extracted from four different volume ranges of whole blood samples, 200, 100, 50 and 200 µl and was eluted in 200, 100, 50 and 50 µl of elution buffer respectively (referred to as experiments 1, 2, 3 and 4 respectively). Phosphate Buffered Saline (PBS) buffer was added to samples that contained less than 200 µl whole blood to bring the final volume to 200 µl as recommended by the manufacturer. Extraction procedure for each volume being tested was performed in duplicate for both ME and EZ methods. Genomic DNA samples (from each of the duplicate extraction) were analyzed in 4 replicates using multiplex qPCR assay. The mean C_T_ values for PLU assay using DNA extracted by ME or EZ methods were 20.69±0.05, 20.36±0.07, 20.43±0.07,19.32±0.07 and 20.71±0.07, 20.43±0.09, 20.32±0.08 and 18.95±0.03 for experiments 1, 2, 3 and 4 respectively. Both extraction procedures performed equally well for all four different blood volumes tested. As expected, experiment 4, where genomic DNA was extracted from 200 µl whole blood and eluted in 50 µl elution buffer produced CT values that were lower [indicating more template DNA present] compared to the other experiments. For the convenience of sample processing and quantification, genomic DNA used in all the experiments from this point on was extracted using EZ method from 200 µl whole blood and eluted in 200 µl elution buffer.

### Inhibition Studies

Inhibition studies were performed to test and compare the co-purification of PCR inhibitors in samples extracted from whole blood using ME or EZ methods. Real-time PCR experiments were performed as described in the materials and methods section using IC plasmid as the template [with IC F/R primers and IC probe; Table 1] with the following modifications. Each 5 µl reaction contained 1.4 µl of genomic DNA extracted from whole blood using ME or EZ methods and 1 µl of IC plasmid DNA as the template. IC plasmid DNA was tested in two different concentrations, 4 replicates each. Control experiments did not contain genomic DNA sample in the reaction. The mean and standard deviation (STDEV) CT values of all experiments were analyzed. Data shows that there were no differences in performance of the qPCR assay between conditions tested (Table 3). At higher IC plasmid DNA concentration, the mean CT value for experiments containing genomic DNA extracted using ME, EZ methods or control (experiment without extracted genomic DNA) was 23.46±0.26 and at lower IC plasmid DNA concentration, the mean CT value was 28.79±0.23. This data illustrates that extraction of genomic DNA from whole blood sample does not co-purify with substances that inhibit qPCR at the volume tested.

**Table 3:** Inhibition studies to test the co-purification of PCR inhibitors.

	ME High	ME Low	EZ High	EZ Low	Control High	Control Low
**Mean CT**	23.4	28.86	23.65	28.92	23.32	28.5
**STDEV**	0.293	0.071	0.111	0.074	0.265	0.141

Two different concentrations of IC DNA were used as the DNA template in the qPCR reactions to test the co-purification of PCR inhibitors in samples extracted from whole blood using ME or EZ methods; High DNA concentration or Low DNA concentration. Control experiments did not contain genomic DNA in the reaction. Each column shows the method which the genomic DNA present [or not for the controls] was extracted [ME or EZ] and amount of IC plasmid present [High or Low].

### Determination of Most Optimal Reaction Volume Required for qPCR Assay

**Figure 1:**
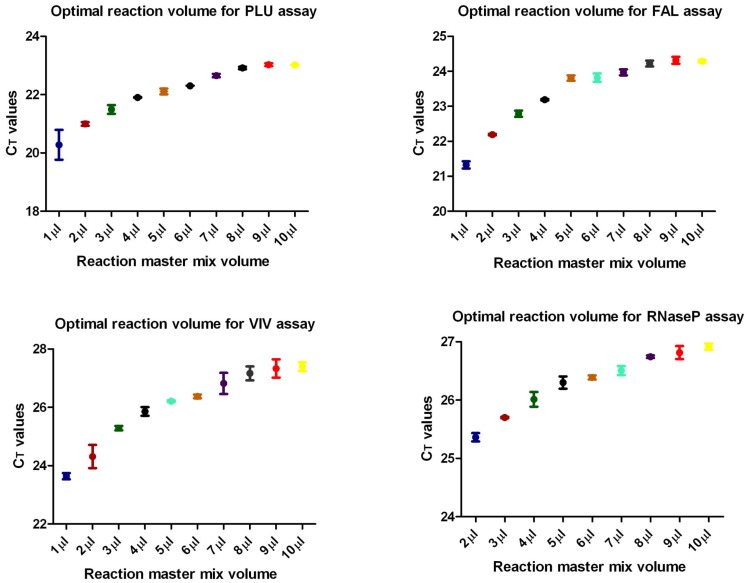
Titration of reaction master mix volume in qPCR reaction. Multiplex qPCR reactions were set-up that contained descending reaction master mix from 10 µl to 1 µl and 1 µl DNA template in each reaction. Experiments were performed in total replicates of 8. All the four targets in the multiplex qPCR assay were analyzed simultaneously. There was a negative correlation between reaction master mix volume and assay sensitivity. As the volume of reaction master mix increased, the sensitivity of the qPCR decreased with 1 µl reaction master mix reaction being the most sensitive for PLU, FAL and VIV assays.

In our previous study [5], qPCR assay was performed by adding 1 µl of template DNA to 9 µl of reaction master mix. The reaction master mix was prepared to a final volume of 20 µl or multiples thereof as needed. To further investigate if the volumes of reaction master mix could be further optimized, a volume titration was performed starting at 10 µl to 1 µl reaction master mix with 1 µl of template DNA used in each reaction. The template DNA used in these experiments contained *P. falciparum* and *P. vivax* genomic DNAs. This sample was prepared by mixing *P. falciparum* and *P. vivax* clinical sample into uninfected fresh whole blood which was then extracted as described using EZ method. Real-time PCR experiments were performed in replicates of 4, and repeated on two separate occasions bringing the number of total replicates performed to 8. All the four targets in the multiplex qPCR assay were analyzed. Surprisingly, for PLU, FAL and VIV assays, the 1 µl reaction master mix (2 µl total reaction volume) was the most efficient with exception of RNaseP assay which did not work (Figure 1). The 2 µl reaction master mix assays performed superiorly as well, with an overall CT values slightly better than the rest of the reactions. In general, data showed a trend where as the reaction volume increased, qPCR assays became slightly less efficient for all the assays with a plateau being reached at reaction master mix of 8 µl. The amplification plots of all the reactions were smooth and looked similar in all the different reaction volumes tested. To further test the importance of molar concentrations of the reactions, starting at 5 µl down to 1 µl reaction master mixes, water was added to bring the final reaction master mix to 10 µl. One microliter DNA template was used in each reaction. Real-time PCR assays were completely compromised with most of the reactions failing to amplify (data not shown).

### Performance of the Absolute qPCR Assays

PLU, FAL and VIV plasmids were used to determine the performance of each absolute qPCR assay. The efficiencies and precision of each replicate assay was evaluated. To determine the efficiency, each plasmid DNA was 5-fold serially diluted 5 times and analyzed in 3 replicates. The slope and the R^2^ values of each curve were used to evaluate the efficiency of each assay whereas STDEV of each replicate was used to evaluate the assay precision. All the absolute qPCR assays performed with efficiency of more than 94%, R^2^ values were 0.99 or greater and the STDEV of each replicate was <0.167.

### Quantification of Absolute qPCR Assay in Terms of Parasite/µl

**Figure 2:**
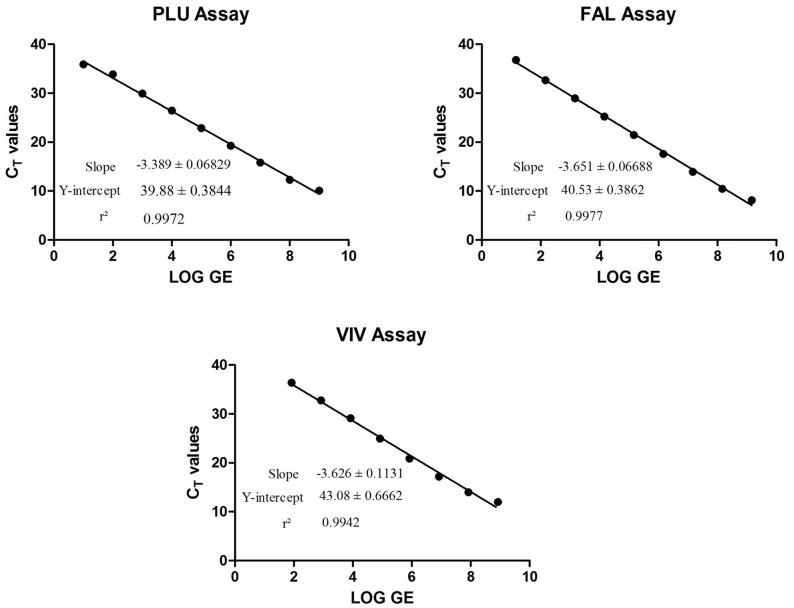
Linear regression plots for absolute qPCR assays. Real-time PCR assays were performed using plasmid DNAs for each assay. Plasmid DNA was 10-fold serially diluted at each point and ran in 4–8 replicates. A linear regression plot was generated using GraphPad Prism. The slope, the Y-intercept and the r^2^ value were determined. Data shown confirms that these assays perform with high efficiencies.

In absolute quantification, sample concentration is expressed in terms of genomic equivalence (GE) or copy numbers. However, for malaria, parasite density is mostly expressed as parasite/µl, based on parasite density as determined by microscopy. It is important therefore that when performing absolute qPCR for malaria, parasite density is expressed in terms that makes clinical sense [and/or other application] and is based on the gold standard for malaria diagnosis which is microscopy. Here, an objective was laid out to determine the amount of GE that is equivalent to parasites/µl. The CT values obtained from absolute and relative qPCR assays were correlated to determine the amount of GE [plasmid DNA] that is equivalent to parasite/µl. NAT assays was used for relative quantification of *Plasmodium spp.* and *P. falciparum* assays whereas *P. vivax* clinical samples were used for relative quantification of the *P. vivax* assay. The parasite density of the NAT assays was determined as described above. For analysis of *P. vivax* parasite density using the VIV assay, 5 clinical samples with known parasite densities as determined by expert microscopists were used. Real-time PCR assays were performed for all the three assays using either serially diluted plasmid DNA (absolute qPCR, as shown in Table 4) or genomic DNA (relative qPCR). To estimate the amount of GE that is equivalent to parasites/µl from the relative qPCR assay, the CT values obtained from relative qPCR assays were interpolated as unknowns from the linear regression standard curve of the absolute qPCR assays to obtain equivalent GE (Figure 2). The amount of GE that corresponds to or is equivalent to parasite density in parasites/µl was estimated based on averages obtained from multiple dilutions for NAT assays and 5 *P. vivax* clinical isolates that had been serially diluted. For the PLU absolute qPCR assay, 10.05 GE corresponds to 1 parasites/µl or 1 GE is equivalent to 0.1 parasites/µl; for the FAL absolute qPCR assay, 3.55 GE correlates to 1 parasites/µl or 1 GE is equivalent to 0.281 parasites/µl; and for the VIV absolute qPCR assay, 7.88 GE corresponds to 1 parasites/µl or 1 GE is equivalent to 0.127 parasites/µl.

**Table 4:** Absolute qPCR CT values obtained vs. the genomic equivalence used.

Sample Name	Avg C_T_	Genomic Equivalence	Sample Name	Avg C_T_	Genomic Equivalence	Sample Name	Avg C_T_	Genomic Equivalence
PLU-1	10.086	1003666666.67	FAL-1	8.109	1459878787.88	VIV-1	11.99	843992424.24
PLU-2	12.267	100366666.67	FAL-2	10.454	145987878.79	VIV-2	13.972	84399242.42
PLU-3	15.827	10036666.67	FAL-3	13.92	14598787.88	VIV-3	17.19	8439924.24
PLU-4	19.235	1003666.67	FAL-4	17.569	1459878.79	VIV-4	20.854	843992.42
PLU-5	22.85	100366.67	FAL-5	21.462	145987.88	VIV-5	24.952	84399.24
PLU-6	26.447	10036.67	FAL-6	25.224	14598.79	VIV-6	29.13	8439.92
PLU-7	29.907	1003.67	FAL-7	28.939	1459.88	VIV-7	32.744	843.99
PLU-8	33.858	100.37	FAL-8	32.665	145.99	VIV-8	36.389	84.40
PLU-9	35.882	10.04	FAL-9	36.788	14.60	VIV-9	Und	8.44
PLU-10	Und	1.00	FAL-10	Und	1.46	VIV-10	Und	0.84
NTC	Und		NTC	Und		NTC	Und	

Data showing the mean C_T_ values obtained from qPCR assays performed using plasmid DNAs. Plasmid DNAs were 10-fold serially diluted 10-log [times] and ran in 4–8 replicates.

### Determination of Limit of Detection

To establish the Limit of Detection (LoD), plasmid DNAs for each assay were 5-fold serially diluted and qPCR assays performed in 4 replicates. The lowest concentration of plasmid DNAs that yielded positive test results in all the replicates were set as the initial LoD. The initial LoD was used as the base point for the 2-fold dilution series to determine the actual LoD. Real-time PCR assays for each plasmid DNA were performed in 4 replicates and actual LoD was established from the lowest plasmid DNA that yielded positive test results in all the replicates. The GE LoD for PLU, FAL and VIV assays were 2.5, 7.3 and 8.4 respectively. To determine LoD for each assay in terms of parasite/µl, GE LoD was multiplied with parasite/µl of GE [0.1, 0.281 and 0.127 for PLU, FAL and VIV assays respectively] of plasmid DNA established for each assay. The calculated LoD in terms parasite/µl based on GE LoD were 0.25, 2.04 and 1.07 for PLU, FAL and VIV assays respectively. Similar dilution strategy was used to establish LoD using genomic DNA. NAT assays DNA was used to determine LoD for PLU and FAL assays whereas *P. vivax* clinical sample DNA was used to determine LoD for VIV assay. The LoDs for PLU, FAL and VIV assays were 0.31, 2.5 and 1.13 parasite/µl respectively. This data demonstrates GE LoD for the three assays compares very well with LoD established using genomic DNA.

### Comparison of Parasite Densities (Parasite/µl) Obtained by Absolute qPCR to Microscopy

**Figure 3:**
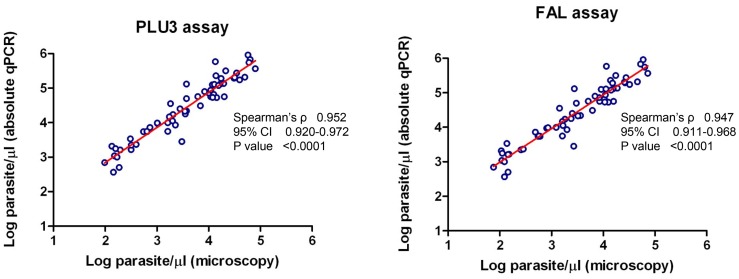
Analysis of parasite densities in clinical samples using absolute qPCR and microscopy. Absolute quantitative qPCR was performed using plasmid DNA as the standard to analyze clinical samples. The log_10_ parasite densities in terms of parasite/µl was determined from qPCR assays and compared to the log_10_ parasite densities as determined by expert microscopist. The correlation coefficient of parasite densities measured using the two methods was calculated using the nonparametric Spearman correlation coefficient. There was a statistically significant correlation between parasite density measured by microscopy and absolute quantitative qPCR.

Parasite densities in terms of parasite/µl were determined in 60 clinical samples (from Kenya) using plasmid DNA as the standard for PLU and FAL assays. These densities were then compared to parasite densities obtained by microscopy. There was statistically significant correlation between parasite densities measured by both methods (Figure 3). The average log_10_ density obtained by microscopy was 4.41 whereas for PLU and FAL assays were 3.46 and 3.54 respectively. We did not have sufficient *P. vivax* samples with well characterized microscopy data to perform similar experiments for the VIV assay.

## Discussion

A multiplex assay that simultaneously detects three *Plasmodium* targets and the human RNaseP gene as an endogenous control is described. The multiplex assay was designed to support on-going vaccine and drug efficacy studies at Walter Reed Army Institute Research (WRAIR) in Silver Spring MD, Southeast Asia and in Africa. Multiplexing qPCR assays increases the likelihood of compromising the efficiency of individual target assays likely due to competitive amplifications and/or interaction of the fluorophores [19]. In this study, we have described a multiplex assay with superior sensitivity of all the individual target assays. This was achieved by testing several fluorophores combinations until the most optimal combination was attained. All the qPCR assays described here performed with high efficiencies of more than 94%, high R^2^ values and very low STDEVs between replicates of each dilution. The acceptable PCR efficiency range is 100% ±10% which is derived from a slope of −3.3±10%. A reaction with lower efficiency will have lower sensitivity. For a PCR assay to be considered 100% efficient, the CT difference between two successive concentrations in a 2-fold dilution is 1. To be able to quantify a 2-fold dilution in more than 99.7% of cases, the STDEV has to be ≤0.167. Data presented here demonstrate that PCR chemistries in all reactions tested are robust.

The preanalytical steps of PCR including DNA extraction are extremely important not only because this is probably the most expensive step of the analysis, but also because the quality of DNA obtained impacts all downstream activities. In routinely performed test(s), DNA extraction must be efficient, convenient and fast. Purification of DNA on EZ1 Advanced XL automated sample purification system takes 17–19 minutes to extract 14 samples using Qiagen EZ1 DNA blood kit. The quality of DNA obtained is the same as that obtained using manual kits. Automation is important because it reduces hands-on time which allows the technician to focus on other steps of setting up qPCR, improving on the overall time of getting results back from a run. It also improves the overall performance and handling of routine PCR assays. Increasing the amount of blood volume extracted does not seem to improve assay sensitivity. Similar observation have been reported elsewhere [11]. In fact, from our experience, when volume of more than 200 µl of blood is used for extraction and the DNA eluted in smaller volumes [such as start with 500 µl of blood and elute the DNA in 50–100 µl elution buffer], the sensitivity of the assay is reduced [data not shown]. Data presented here show that there was no evidence of PCR inhibitors co-purified using either ME or EZ methods. Similarly, use of 1 µl of DNA template is the most optimal [data not shown] compared to using higher volumes of DNA in qPCR reactions.

In this study, we have shown that low volume reactions were more sensitive, with 2 µl total reaction volume being the most sensitive except for RNaseP assay. When the reaction master mix used in qPCR was titrated, there was a negative correlation between total reaction volume and qPCR sensitivity. It is likely that with small volumes, the temperature cycling is more efficient. However, it is important to be cognitive of the fact that reduced reaction volumes maybe more sensitive to volume variation which may affect assay outcome such as the reproducibility and standard deviations of replicate reactions. Such variation may result from pipetting errors or evaporation during the qPCR cycling. In our labs, we have adapted a standard practice of using reaction volumes of 3 or 4 µl to reduce the likelihood of volume variations. Consistence use of 1 µl reactions might require using specialized PCR tubes or 384 well plates that are specifically designed for smaller volumes. Both reaction volume and the concentration of the reactants are critical to ensure success of a low volume qPCR reaction.

Cost is one of the most prohibitive aspects of qPCR especially in resource constrained laboratories in austere locations where malaria is found. As such, cost has inhibited the adoption of qPCR over microscopy as the gold standard method for malaria diagnosis. QuantiFast Probe PCR kit and most other commercial master mix kits recommends using a total reaction volume of 20–25 µl. Excluding the cost of DNA extraction and labor, at the current list prices of master mixes, primers and probes, a singleplex reaction containing total volume of 25 µl costs ∼$1 whereas a reaction containing total volume of 3 µl costs ∼$0.08, more than 90% reduction in cost. A four target multiplex reaction as described here costs ∼$0.11. The reduced costs of qPCR as described makes its application in high through-put qPCR assays advantageous for epidemiological and surveillance studies. It is important to note however that the cost of DNA extraction remains the single most limiting factor. It is extremely important that less expensive DNA extraction methods are developed for PCR to be more affordable and accessible. In this study, DNA was extracted using column based methods. However, large scale field studies often employ less expensive and simpler methods of DNA extraction such as Chelex-100 extraction. If well stored, extracted DNA can be used in numerous PCR experiments which can be argued that it lowers the cost of DNA extraction. It is important however that more active effort and research is directed towards improving the efficiency and lowering the costs of DNA extraction.

Malaria parasite density is expressed in terms of parasite/µl, based on Giemsa-staining of thick and/or thin blood smears. If qPCR assay is going to replace microscopy as the gold standard diagnostic method, it is important that quantification is expressed as parasites/µl. Currently, qPCR assays use relative standard quantification methods to quantify parasite density in a sample where cultures or clinical samples with known parasite density are used. This method depends on accurate preparation of standard DNA every time needed which may involve growing cultures or obtaining clinical sample quantified my an expert microscopist. Such practices are inconvenient, time consuming, expensive and can be source for error. Absolute quantitative qPCR assay present many advantages in accuracy and consistency. Also, Plasmid DNA can be produced in large quantities and if properly handed and stored, it can last for a long time. However, different structural types of standard DNA (circular versus linear) have been shown to affect the quantification and accuracy of qPCR assays. Activities that might affect the structure of plasmid DNA include freeze thawing, pipetting and vortexing. A recent study demonstrated that the linear DNA standards including linearized plasmids, but not the circular plasmid, are more reliable for absolute qPCR [13]. Therefore, it might be important to linearize plasmid DNAs generated in this study to improve their reliability. The sensitivity of the assays in parasite/µl compared well when using either plasmid DNA or genomic DNA. In addition, there was a significant correlation between parasite densities and in terms of parasite/µl established using both methods. However, the average parasite density established using microscopy was higher compared to density established using qPCR, a phenomenon that have previously been observed [11]. To the best of our knowledge, this is the first time that plasmid DNA has been used in quantification of *Plasmodium*.

## Conclusion

Microscopy has remained the gold standard for malaria diagnosis because of its simplicity, affordability and the ability to quantify the parasite density. The shortcomings associated with microscopy have also been extensively published and discussed. However, microscopy is far from being replaced universally as the gold standard for malaria diagnosis especially because it is affordable. As molecular techniques become much more widely used and acceptable as alternate or as confirmatory assays to microscopy, they must meet and exceed qualities and characteristics that have made microscopy popular for as long as it has. All aspects and characteristics of molecular diagnosis must be carefully investigated, and most important, harmonized. Towards this effort, we have presented a multiplex assay which for the first time, absolute quantification of malaria parasite is described. Most important, the parasite quantity is described in parasite/µl, the same way as described when quantified by microscopy or relative qPCR. Using Minimum Information for Publication of Quantitative Real-Time PCR Experiments (MIQE) guidelines as recently published [20], we have also described other qPCR assay characteristics that are important in molecular analysis. The multiplex assay described here can be used as is in areas where both *P. falciparum* and *P. vivax* co-exist in the population such as South East Asia and some parts of Africa such as Ethiopia. However, a subset of the assays reported here could be used in African populations, with the *P.vivax* assay replaced with other relevant diagnostic assays for detection of P. *ovale* and/or *P. malariae*.
